# The teleost fish, blue gourami *Trichopodus trichopterus*, distinguishes the taste of chemically similar substances

**DOI:** 10.1038/s41598-020-64556-6

**Published:** 2020-05-04

**Authors:** Alexander O. Kasumyan, Grigoryi E. Mouromtsev

**Affiliations:** 1 Faculty of Biology, Lomonosov Moscow State University, Moscow, 119991 Russian Federation; 2 Institut Le Rosey, Rolle, 1180 Switzerland

**Keywords:** Ichthyology, Physiology, Zoology

## Abstract

Behavioural approaches permit studies of the functional features of animal gustatory systems at the organism level, but they are seldom used compared to molecular and electrophysiological methods. This imbalance is particularly apparent in studies on fish gustation. Consequently, our notion of taste preferences remains limited in fish, the most numerous and diverse group of vertebrates. The present study aimed to determine whether fish could distinguish the tastes of chemical substances with similar structures and configurations. We performed behavioural trials, where each test substance (L-alanine, glycine, L-cysteine and 9 of their derivatives; 0.1 M) was incorporated into agar pellets, and presented to blue gourami (*Trichopodus trichopterus*). We found that L-α-, L-β-, and D-α-alanine as well as L-cysteine and L-cystine had different palatabilities; and glycine, methyl-glycine, dimethyl-glycine-HCl, trimethyl-glycine, and glycyl-glycine had similar taste qualities. Results show that molecular transformation could shift the palatability of amino acids, which led to changes in the orosensory behaviour of blue gourami. The ability of fish to display different taste preferences for substances, like amino acids and their, derivetives, widely distributed among aquatic organisms, undoubtedly forms the sensory basis for selective feeding, which in turn, reduces the competition for food among sympatric species in natural waters.

## Introduction

Unlike most other sensory modalities, gustation regulates only one type of behaviour – feeding behaviour. Gustation participates in evaluating food quality and in making decisions on the relevance of food to nutritional demands^1^. This highly valuable function prevents consumption of inappropriate or dangerous food in all animals; it also permits them to select the most suitable food out of the vast amount of available and adequate items^2–5^. The morphology and physiology of the animal gustatory system have been studied by many research groups. In recent years, substantial progress in this area has been made, due to the implementation of various new electrophysiological, molecular, genetic, and immunological methods. Different types of taste receptors have been found, and the genes responsible for their formation have been identified. Investigations have revealed the polymorphism and localization of taste receptors within taste bud cells, the mechanisms of specific signal transduction pathways, and the processing of these signals in nerve centres^6–13^. In marked contrast, relatively few studies have investigated gustatory function at the organism level, which could permit estimations of animal taste preferences, evaluations of the palatability of variable substances and food items, and determinations of how these abilities might depend on the conditions of the animals and the influences of many internal and environmental factors^14–17^. Moreover, methods based on behavioural tests permit the detailed study of the molecular and physiological properties of taste reception^18–20^.

An imbalance in the use of different approaches has been evident in studies on the gustatory system of fish, despite the fact that the fish gustatory system is more highly developed than in other vertebrates^21^. Suffice it to say that, in many fish, taste buds are distributed throughout the oral and gill cavities and over the entire body surface and fins, including the caudal fin. The total number of taste buds in fish might be close to one million, which is several magnitudes greater than the numbers found in terrestrial vertebrates^22–24^. Additionally, taste sensitivity in fish exceeds that of all other vertebrates^25^. Due to these and other features, the zebrafish, *Danio rerio*, and several other fish species have recently become models in studies on the fundamental aspects of animal taste reception^12,18,26^. Nevertheless, some important features of taste reception in fish, the largest and most variable group of vertebrates, are weakly developed or have not been studied. One unresolved question is whether fish can distinguish taste quality among different chemical substances that have similar formulae and spatial configurations.

Studies based mainly on electrophysiological data have indicated that molecular changes in substances might have significant effects on their efficacy as gustative stimuli for fish. It was shown that the addition, substitution, or transfer of functional groups in a molecule, or the use of different stereoisomers led to different responses, in terms of response amplitudes and other parameters of evoked electrophysiological responses^27–31^. Indirectly, these data indicated that a modification in a given molecule could influence the palatability of the substance to fish. However, it remains to be determined what kind of influence might be conferred and how much the palatability might change.

It should be emphasised that the recordings of nervous activity do not tell about the animals’ reaction towards a specific chemical. There are many discrepancies between the sensory input revealed by electrophysiological means and the behavioural output, which emphasise that palatability is not synonymous with excitability in the taste system (see for review^32^). For example, recordings from the palatine nerve in Atlantic salmon *Salmo salar* showed that of the few amino acids tested, all were non-stimulatory with the exception of L-proline. However, L-proline was an indifferent taste substance in bioassays^33^. In Arctic char *Salvelinus alpinus* L-glutamic acid was found to be the most palatable substance in behavioural assays but had no effect in electrophysiological studies. In contrast, L-proline, and L-alanine were the most effective taste stimuli in electrophysiological study for Arctic char but were classed as neutral taste substances in behavioural assays^34,35^. Hydroxy-L-proline, L-alanine and betaine evoked intense neural responses in rainbow trout *Oncorhynchus mykiss* palate nerve but were not palatable taste substances for this fish^28,36,37^. Adenosine-5′-monophospate, inosine-5′-monphosphate, urindine-5′-monophosphate, and adenosine-5′-diphosphate were not palatable for puffer *Fugu pardalis* in spite of their marked stimulatory effectiveness on the lip chemoreceptors of this fish^38^.

The present study aimed to compare the palatability of free amino acids and their derivatives, including stereoisomers and dipeptides, in the blue gourami, *Trichopodus trichopterus*, closely related to the pearl gourami *T. leerii* which were a convenient fish for this type of experiment^39^. To solve this task alanine, glycine and cysteine were selected based on previous studies showing high efficiency these amino acids as taste stimuli in many fish species^15,16,21,25,29,32,36^. We also addressed the question of whether modifications in the molecular structures of taste substances could initiate changes in fish behaviour during their orosensory testing of food flavoured with these substances.

## Methods

### Fish maintenance and training

Nine fish were purchased from a Moscow pet shop for the experiments. The average body length was 54 mm (range 45–58 mm) and the average weight was 2.26 g (range 1.72–2.76 g). Fish were maintained separately in rectangular aquaria with a water volume of 7 l. To prevent visual contact, the lateral walls were opaque. The water temperature was maintained at a stable 24 °C with aquatic heaters (AquaEL Easyheater EH-25W; China). The illumination levels followed environmental fluctuations common for July, at 55° 45′N. Gravel was not added. Fish were fed *ad libitum* live mosquito larvae (Chironomidae) once per day after the experiments ended (around 16-00 p.m.), and then half of the water in each aquarium was replaced with fresh water (temperature 24 °C; conductivity 600–700 µS/cm; pH 6.4–6.7; salinity 0.03 g·l^−1^).

**Figure 1:**
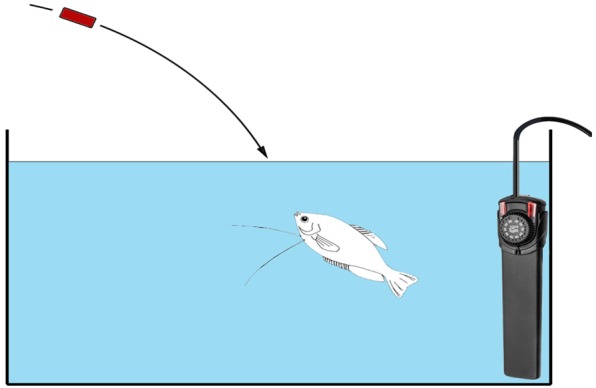
Experimental setup: test tank (7 l) equipped with an electric heater (25 W); a single blue gourami was first trained to grasp red-coloured, standard-sized pellets.

Before the experiments, the fish were taught to catch live mosquito larvae, presented one by one, then to catch pellets of agar gel (2%) that contained water extract of mosquito larvae (175 g l^−1^) and the red dye, Ponceau 4R (5 мкМ; Chroma-Gesellschaft Schmidt Gmbh; Germany). After completing this training (2–3 days), the fish grasped the presented pellets within several seconds of their falling into the water (Fig. 1).

### Tested substances and agar pellets

For experiments, we used pellets that contained red dye and 0.1 M of one of the tested substances (amino acids and their derivatives, Sigma, Merck). All substances were classified, by convention, into one of three groups. The L-α-alanine group included L-α-alanine, L-β-alanine, and D-α-alanine. The glycine group included glycine, methyl-glycine (=sarcosine), dimethyl-glycine-HCl, trimethyl-glycine (=betaine), glycyl-glycine, glycolic (hydroxyacetic), and acetic acids. The L-cysteine group included L-cysteine and L-cystine. The control pellets contained only dye. Pellets that contained the mosquito larvae extract were used to control the motivation of fish to feed. To prepare the extract, mosquito larvae were homogenized in a ceramic mortar and the homogenate was mixed with distilled water in the proportion of 175 g·l^−1^ and then was centrifuged (7800 rpm, 15 min) after extraction for 5 min at 20 °C. Obtained supernatant was added into agar gel solution.

Immediately before offering the pellets to fish, the pellets were excised from the gel with a stainless-steel tube, which produced pellets of 1.35 mm in diameter and 4.0 mm in length. All test substances are termal stable^40^ and were added to a hot agar solution (60–70 °С). After cooling, the agar gel, flavoured with amino acids and derivates, was stored in a refrigerator at +5 °С for no more than two weeks, and the agar gel flavoured with the water extract of mosquito larvae was stored for no more than three days.

### Experiments

In every experiment, we offered one pellet to the fish, and we recorded five paramethers of fish response: (i) the time between the pellet falling into the water and the first grasp by a fish; (ii) the number of pellet grasps during the entire experiment; (iii) the time that the pellet was retained in the fish’s mouth for the first capture; (iiii) the total time that pellets were retained in the mouth for all grasps during the entire experiment; (iiiii) and the ingestion or refusal to ingest the pellet throughout the experiment (consumption). The time between the pellet falling into the water and the first grasp by a fish was recorded visually. To record the duration of the different experimental stages, we used an electronic hand-held stopwatch (JUNSO JS-710, China; accurate within 0.01 s). Every experiment lasted about 1 min and ended with pellet ingestion or with the refusal to consume. Refusal to consume was judged based on the behaviour of the fish (i.e., stopped biting, lost interest in the pellet and swam away from it). Infrequently, pellets were not grasped within 1 min or ingestion could not be determined because the pellet was destroyed by the fish and formed numerous small fragments; these data were not included in the analysis. Pellet or fragments that were not ingested were removed from the aquarium immediately after the end of each experiment.

Experiments were started at 10-00 a.m. and during the day each fish were offered by the one pellet of each 14 types of pellets, including control one, in a random sequence. The intervals between successive experiments with the same fish were 15–20 min. In total, 5–6 repetitions were performed for each chemical substance on each fish. All experiments were carried out blind. One person who gave coded number for each gel prepared all agar gels. Another person tested pellets cut from these gels. All measurements made by the same observer.

The authors confirm that all methods were carried out in accordance with relevant guidelines and regulations and were approved by the Lomonosov Moscow State University Bioethic Committee (Protocol # 108-0).

### Statistics

In all, we carried out 702 experiments. Chi-squared tests were conducted to detect statistically significant difference in consumption of flavoured pellets in relation to control ones, and the Mann-Whitney *U* tests were conducted to detect statistically significant difference for other four parameters of fish response to flavoured pellets in relation to control ones. To evaluate the palatability of substances, we calculated the index of palatability with the following formula: *Ind*_pal_ = [(*R* – *C*)/(*R* + *C*)] × 100, where *R* was the proportion (%) of pellets ingested that contained the tested substance, and *C* was the proportion (%) of control pellets ingested^32^. To evaluate the relationship between all parameters of fish behavioural response to flavourad pellets the Spearman rank correlation coefficient (*r*_*s*_) was calculated.

## Results

Of the 12 substances used for testing, only L-α-alanine and glycolic acid elicited a substantial increase in pellet consumption. The efficiency of L-α-alanine consumption was close to that of the mosquito larvae extract. D-α-alanine, glycine, trimethyl-glycine, and L-cystine did not influence the consumption of pellets. The other 6 substances had an aversive effect; i.e., their presence in pellets lead to a considerable reduction in consumption. The most pronounced negative influence was observed with acetic acid, which reduced the consumption by nearly 5 fold, relative to the control (Table 1).

**Table 1:** Behavioural taste responses of blue gourami *Trichopodus trichopterus* to pellets of agar gel (2%) flavoured with free amino acids or their derivatives (0.1 М).

Taste substances	Latent period, s	Pellet consumption, %	Index of palatability	Number of grasps over the entire trial	Pellet retention time, s:		Number of trials
					after the first grasp	over the entire trial	
L-α-Alanine	4.27 ± 0.42	92.0 ± 3.9***	31.4	1.20 ± 0.06***	7.74 ± 0.50***	8.51 ± 0.46***	50
D-α-Alanine	3.62 ± 0.43*	40.8 ± 7.1	−8.1	2.49 ± 0.23	4.71 ± 0.66	8.03 ± 0.79**	49
L-β-Alanine	3.35 ± 0.23	25.5 ± 6.2*	−30.6	2.16 ± 0.19	2.74 ± 0.28	4.71 ± 0.56	51
Glycine	4.46 ± 0.36	34.0 ± 6.8	−17.1	1.82 ± 0.16	4.45 ± 0.51	5.94 ± 0.54	50
Glycyl-glycine	3.68 ± 0.30	20.0 ± 5.7**	−41.2	2.40 ± 0.27	1.95 ± 0.24***	3.50 ± 0.42*	50
Methyl-glycine (=sarcosine)	5.08 ± 0.87	20.0 ± 5.7**	−41.2	2.20 ± 0.22	2.51 ± 0.30	4.30 ± 0.53	50
Dimethyl-glycine-HCl	5.40 ± 1.05	25.5 ± 6.2*	−30.6	2.25 ± 0.22	3.16 ± 0.45	7.99 ± 0.86*	51
Trimethyl-glycine (=betaine)	4.23 ± 0.91*	30.0 ± 6.5	−23.1	2.24 ± 0.22	3.80 ± 0.26	6.25 ± 0.78	50
Glycolic acid	5.36 ± 0.51	74.0 ± 6.3**	21.3	1.88 ± 0.23	5.78 ± 0.65**	7.77 ± 0.71**	50
Acetic acid	4.68 ± 0.51	10.0 ± 4.3***	−65.5	2.94 ± 0.40	1.57 ± 0.16***	4.09 ± 0.73**	50
L-Cysteine	4.25 ± 0.76	28.0 ± 6.4*	−26.3	3.08 ± 0.33*	3.19 ± 0.44	5.21 ± 0.52	50
L-Cystine	4.64 ± 0.70	66.0 ± 6.8	15.8	2.40 ± 0.40	5.83 ± 0.62***	8.71 ± 0.66***	50
Mosquito larvae extract^a^	4.63 ± 0.56	100.0 ± 0.00***	35.1	1.14 ± 0.07***	7.41 ± 0.39***	7.72 ± 0.40***	51
Control (blank pellets)	5.15 ± 0.55	48.0 ± 7.1	—	2.16 ± 0.19	3.28 ± 0.39	4.89 ± 0.44	50

^a^Concentration of water extract of mosquito larvae in pellets = 175 g. l^−1^. Values are mean ± s.e.m. *P < 0.05, **P < 0.01, ***P < 0.001 relative to control (χ^2^ test – for pellet consumption; the Mann-Whitney *U* test – for others).

**Figure 2:**
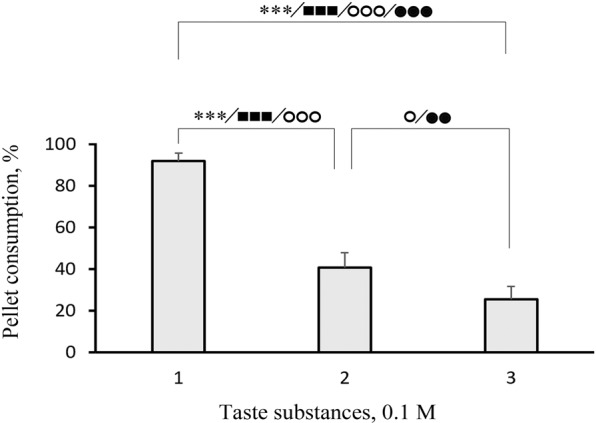
Behavioural taste responses observed in blue gourami *Trichopodus trichopterus* to pellets of agar gel flavoured with 0.1 М (1) L-α-alanine, (2) D-α-alanine, or (3) L-β-alanine. Significant differences relative to control (blank pellets) are indicated for pellet consumption (asterisks; χ^2^-test), the number of grasps (filled squares), and pellet retention times for the first grasp (open circles) and for all grasps over the entire trial (filled circles; Mann-Whitney *U* test). One, two, and three symbols indicate *P* < 0.05, *P* < 0.01, and *P* < 0.001, respectively. Whiskers are the s.e.m.

**Figure 3:**
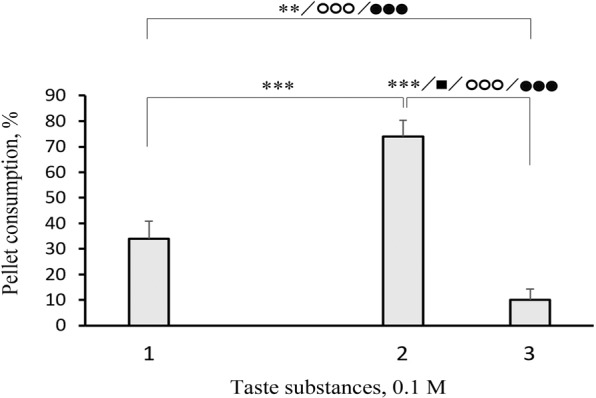
Behavioural taste responses observed in blue gourami *Trichopodus trichopterus* to pellets of agar gel flavoured with 0.1 М (1) glycine, (2) glycolic acid, and (3) acetic acid, Significant differences relative to control (blank pellets) are indicated for pellet consumption (asterisks; χ^2^-test), the number of grasps (filled squares), and pellet retention times for the first grasp (open circles) and for all grasps over the entire trial (filled circles; Mann-Whitney *U* test). One, two, and three symbols indicate *P* < 0.05, *P* < 0.01, and *P* < 0.001, respectively. Whiskers are the s.e.m.

**Figure 4:**
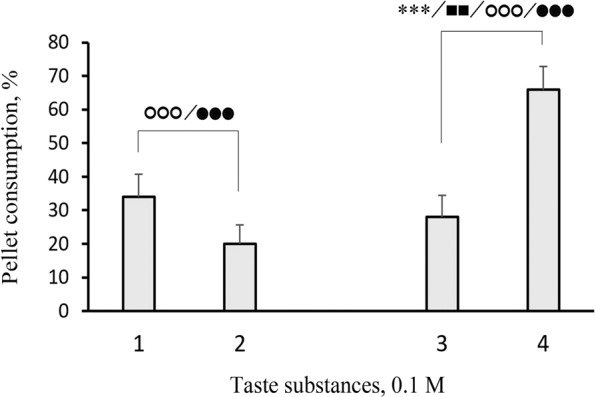
Behavioural taste responses observed in blue gourami *Trichopodus trichopterus* to pellets of agar gel flavoured with 0.1 М (1) glycine, (2) glycyl-glycine, (3) L-cysteine, (4) and L-cystine. Significant differences relative to control (blank pellets) are indicated for pellet consumption (asterisks; χ^2^-test), the number of grasps (filled squares), and pellet retention times for the first grasp (open circles) and for all grasps over the entire trial (filled circles; Mann-Whitney *U* test). One, two, and three symbols indicate *P* < 0.05, *P* < 0.01, and *P* < 0.001, respectively. Whiskers are the s.e.m.

**Figure 5:**
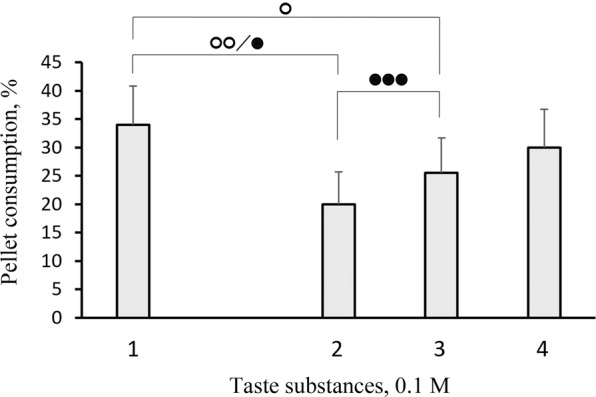
Behavioural taste responses observed in blue gourami *Trichopodus trichopterus* to pellets of agar gel flavoured with 0.1 М (1) glycine, and its methyl derivatives, (2) methyl-glycine, (3) dimethyl-glycine-HCl, (4) and trimethyl-glycine. Significant differences relative to control (blank pellets) are indicated for pellet consumption (asterisks; χ^2^-test), the number of grasps (filled squares), and pellet retention times for the first grasp (open circles) and for the entire trial (filled circles; Mann-Whitney *U* test). One, two, and three symbols indicate *P* < 0.05, *P* < 0.01, and *P* < 0.001, respectively. Whiskers are the s.e.m.

A comparison of fish reactions to the control and other substances revealed that the consumption and other parameters of the response could differ substantially for substances in the same class. Indeed, we found considerable differences between the consumption of pellets that contained L-α-alanine, L-β-alanine, and D-α-alanine (Fig. 2); between the consumption of pellets that contained glycine, glycolic acid, and acetic acid (Fig. 3); and between the consumption of pellets that contained L-cysteine and L-cystine (Fig. 4). However, we found no significant difference between the consumption of pellets that contained glycine and pellets that contained substances of the same class, including methyl-glycine, dimethyl-glycine-HCl, trimethyl-glycine, and glycyl-glycine (Fig. 5).

The pellets of all types were grasped by fish very soon, on average 3–5 s, after they dropped into the water (the latent period). Fish frequently rejected the grasped pellet, but grasped it repeatedly. This acts occurred in excess of 50% of experiments, for 10 out of the 14 types of pellets used. The number of repeated grasps during the experiment typically did not exceed 6–8, but in some trials, fish performed 11–16 repetitions (i.e., L-cysteine, L-cystine, glycyl-glycine, glycolic acid, and acetic acid). Repeated grasps were recorded most frequently for pellets flavoured with L-cysteine, and least frequently for pellets flavoured with L-α-alanine and the mosquito larvae extract (Table 1)

**Figure 6:**
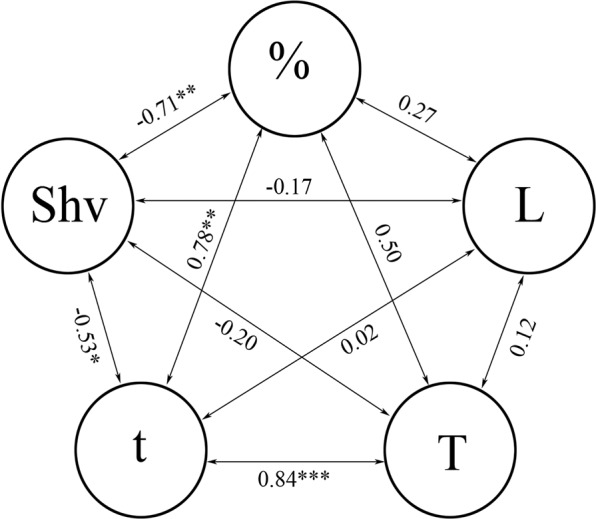
Spearman rank correlations between different parameters of behavioural taste responses to pellets of agar gel flavoured with free L-amino acids and their derivatives observed in blue gourami *Trichopodus trichopterus*. L: latency in the response to the presented pellet; %: proportion of pellets consumed; Shv: number of pellet grasps; t and T: pellet retention times for the first grasp and for all grasps over the entire trial, respectively. *P < 0.05; **P < 0.01; ***P < 0.001.

The retention times also varied. The shortest retentions lasted 0.5–0.7 s and the longest retentions lasted 20–24 s. The average retention times for different pellets varied from less than 2 s to nearly 8 s for the first grasp, and from 3.5 s to almost 9 s for all grasps during the entire experiment (Table 1). A considerable correlation was found between the pellet consumption and the pellet retention time and the number of repeated grasps (Fig. 6).

## Discussion

Our study results revealed that some chemical substances, though similar in structure, had different taste qualities to the blue gourami. Moreover, we found that different configurations of a molecule, caused by chirality or small structural modifications, could lead to dramatic changes in palatability. In blue gourami, the feeding responses stimulated by the L- and D- isomers of α-alanine differed by more than two-fold. The same difference in palatability between the α-alanine stereoisomers was found previously in experiments with the three-spined stickleback, *Gasterosteus aculeatus*^41^. In fact, electrophysiological experiments in various fish species showed that many L-amino acids exhibited higher stimulation efficiencies compared to their D-forms^27–31,42,43^. At the same time, for other amino acids, stronger electrical responses were stimulated by D-isomers than by L-isomers^44–46^. Interestingly, three-spined sticklebacks consumed equal amounts of pellets flavoured with L- and D-glutamic acid, unlike their consumption of pellets flavoured with L- and D-alanine^41^. In rainbow trout (*Oncorhynchus mykiss*) and juvenile common eel (*Anguilla anguilla*), feeding behaviour was stimulated by a mixture of  L-, but not D-amino acid isomers^47,48^. These data indicated that a higher efficiency of L-isomers over D-isomers should not be considered a general rule for all amino acids. However, it is beyond question that the different amino acid stereoisomers might have different palatabilities for fish, for other animals, and for humans^49–52^.

The transfer of an amino group to a new position was associated with a large change in palatability. Indeed, the consumption of pellets with L-β-alanine was nearly 4-fold lower than the consumption of pellets with L-α-alanine. These results were consistent with the results of electrophysiological experiments with the α- and β- forms of alanine^28,29,42,43^. Replacing the amino group in the glycine molecule with a hydroxyl group or a hydrogen atom led to similar strong shifts in taste attractiveness. Blue gourami was indifferent to the taste of glycine (aminoacetic acid); however, after replacing the –NH_2_ in glycine (aminoacetic acid) with –OH to convert it to glycolic acid (hydroxyacetic acid), it became an attractive taste to blue gourami. In contrast, replacing the –NH_2_ in glycine with a hydrogen to form acetic acid had the opposite effect; palatability was reduced almost by 3.5 fold. Indeed, acetic acid and glycolic acid differed in palatability by more than 7 fold (Fig. 3). In the marble goby (*Oxyeleotris marmorata*), these three substances were ranked according to palatability, as follows: glycolic acid > acetic acid >> glycine^16^. Replacing the hydrogen in L-proline with a hydroxyl group to form hydroxy-L-proline weakened the electrophysiological response in common carp (*Cyprinus carpio*) and rainbow trout^28,29,53^. In contrast, in the Japanese eeltail catfish (*Plotosus japonicus*), no great difference was observed between the response to L-proline and the responses to hydroxy-L-proline and dihydroxy-L-proline (i.e., proline with two hydroxyl radicals)^31^. These comparisons indicated that molecular changes in taste substances could have different effects on palatability for different fish species.

However, successive replacements of the hydrogen atoms in the amino group of glycine with methyl groups (–CH_3_) did not substantionally influence the palatability of the amino acid. Despite these considerable molecular transformations, we observed no considerable differences in the consumption of pellets flavoured with glycine, methyl-glycine, dimethyl-glycine-HCl, and trimethyl-glycine in any pairwise comparisons. Nevertheless, we noted the presence of one tendency: as more methyl groups were placed in the glycine molecule, the less they influenced palatability (Fig. 5). A previous study tested the electrical activity evoked by amino acids in the mixed facial and trigeminal nerve branch, which innervates taste buds and/or solitary chemosensory cells on the surface of the maxillary barbels of Japanese eeltail catfish. The effects of glycine derivatives were ranked according to their effects on electrical activity as follows: trimethyl-glycine > methyl-glycine > dimethyl-glycine-HCl > glycine^31^. Trimethyl-glycine (=betaine) is found in many animals that fish feed (e.g., worms, crustaceans, molluscs, etc.)^54–57^; Therefore, betaine is frequently used as an appropriate stimulus in studies on taste reception. Betaine has an attractive taste for some fish species, such as fugu *Fugu (Takifugu) pardalis* and common sole (*Solea solea*)^58,59^. In contrast, many other species are indifferent to the taste of betaine, including blue gourami and various sturgeons of the genus *Acipenser*, the red sea bream, *Pagrus* (*Chrysophrys) major*, the European plaice, *Pleuronectes platessa*, rainbow trout, Chinook salmon (*Oncorhynchus tshawytscha*), bigmouth bass (*Micropterus salmoides*), and crucian carp (*Carassius carassius*)^36,60–65^. Undoubtedly, it is precisely these interspecies differences in betaine palatability that determine how glycine and its methylated forms were ranked in efficiency as gustatory stimuli for blue gourami and the Japanese eeltail catfish.

We found that replacing a hydrogen atom in the amino group of glycine with another glycine molecule led to small changes in taste quality. Although this glycine dipeptide had an aversive taste (fewer glycyl-glycine pellets were consumed by fish compared to control pellets), the difference between glycine and glycyl-glycine consumption was statistically insignificant (Fig. 4). Thus, we concluded that glycine and glycyl-glycine had the same palatability for blue gourami. Few previous studies compared the palatability of dipeptides and amino acids for fish and most data were obtained with electrophysiological experiments. Those studies showed that the excitations elicited in the taste system by various dipeptides were always weaker than the excitations elicited by the pure amino acids^42,66^. However, in humans, most of the 46 tested dipeptides, including glycyl-glycine, had a bitter or unpleasant taste, unlike the taste of pure glycine, which was perceived as sweet^67,68^.

Interestingly, L-cystine, which consists of two molecules of L-cysteine, had twice the palatability of a single L-cysteine amino acid (*P* < 0.001; Fig. 4). The presence of L-cysteine in pellets reduced their consumption compared to controls. However, the presence of L-cystine caused the opposite effect, closer to stimulating consumption (*P* = 0.069 compared to control). L-cystine is not formed by the interaction between the amino- and carboxyl groups of two L-cysteine molecules; instead, it is formed by oxidative dimerization, and a disulfide bond forms between the thiol groups. Thus, unlike glycyl-glycine, L-cystine is not a dipeptide; it is a basic amino acid that contains two amino and two carboxyl groups in one molecule. The closely-related pearl gourami fish showed aversive responses to the diamino acids, L-arginine, and L-lysine, and the dicarboxylic acids, L-glutamic acid and L-aspartic acid^41^. In contrast, blue gourami fish were indifferent to the tastes of these amino acids, except for aspartic acid, which increased pellet consumption (unpublished data). L-cystine is frequently used as supplement in artificial fish feeds instead of methionine^69,70^. However, information is very limited on L-cystine as a taste stimulus for fish. L-cystine was reported to reduce feeding activity in the red sea bream, *Pagrus major*^60^. Moreover, L-cystine did not evoke electrophysiological responses in the external taste receptors of Hawaiian goatfish (*Parupeneus porphyreus*)^71^.

The present study revealed that different molecular configurations and structural alterations shifted the palatability of amino acids, and led to clearly defined changes in the feeding behaviour of fish. For example, blue gourami repeated orosensory testing half as often and spent much less time in the oral evaluation of feeding adequacy for pellets with L-β-alanine compared to pellets with L-α-alanine. Moreover, blue gourami grasped L-cysteine pellets 1.3-fold more often and retained them for nearly 2-fold less time than the L-cystine pellets. In examining the sequence of glycine-glycolic acid-acetic acid, we found substantial changes, both in the consumption of pellets and in all the other parameters of the behavioural response. These findings could be explained by the significant correlations we found among the measured parameters (Fig. 6). We found no significant correlations for the parameter “latency of reaction”, which could be explained by the strict standardization of all visual cues in the pellets; they were equivalent in size, shape, and colour. In some instances, some parameters, like the number of repeated grasps and the duration of pellet retention, were more sensitive to changes in molecular structure than the pellet consumption parameter. For example, glycine and the dipeptide glycyl-glycine did not differ in pellet consumption, but they substantially differed in pellet retention for the first grasp (by 2.3 fold) and for all grasps during the experiment (by 1.7 fold; Fig. 4).

In conclusion, our results corroborated the notion that fish have the ability to finely differentiate among similar chemical substances, based on taste. Modifications in the composition or configuration of an amino acid molecule could lead to notable changes in its palatability; some changes improved its taste attractivity, and others converted it into an aversive taste. These differences were observed for amino acid stereoisomers and for amino acid derivatives that had the amino group moved to a different position, removed, or replaced with another functional group. However, not all structural modifications in the molecules affected the palatability of the amino acids; for example, a methylation of the amino group or the formation of a dipeptide did not cause palatability changes, or they caused only weak changes, at least for blue gourami.

This study was realized by testing particular amino acids and their derivatives as examples of how chemical structure can influence taste. Based on the existing, but limited data from this study and from the available literature, we can speculate with some certainty that, for various amino acids, identical molecular alterations will not lead to similar changes in the strength or sign of their taste attractiveness. We could not exclude the possibility that specific alterations in different amino acids might cause the opposite sensory effects or might not cause any change at all. Considering species-specific taste preferences among different fish for amino acids and other substances^32^, we might expect that, in different species of fish, the same amino acid derivatives could stimulate different responses.

The high potentiality of fish to distinguish the taste of chemically similar substances, like amino acids and their derivates, widely distributed among aquatic organisms, undoubtedly plays an important role in the consumption of some available food items and the avoidance of others. Food differentiation and selective feeding is common characteristic which really observed in various fish that inhabited natural bodies of water^72–77^. The ability to differentiate taste preferences for similar substances forms the sensory basis for selective feeding, which in turn, reduces the competition for food among sympatric fish species in natural waters.
